# Acacia Fiber Protects the Gut from Extended-Spectrum Beta-Lactamase (ESBL)-Producing Escherichia coli Colonization Enabled by Antibiotics

**DOI:** 10.1128/msphere.00071-22

**Published:** 2022-05-18

**Authors:** Marlène Maeusli, Nicholas Skandalis, Bosul Lee, Peggy Lu, Sarah Miller, Jun Yan, Yuli Talyansky, Rachel Li, Zeferino Reyna, Noel Guerrero, Amber Ulhaq, Matthew Slarve, Ioannis Theologidis, Brad Spellberg, Brian Luna

**Affiliations:** a Department of Medicine, Keck School of Medicine, University of Southern California (USC), Los Angeles, California, USA; b Department of Molecular Microbiology and Immunology, Keck School of Medicine, University of Southern California (USC), Los Angeles, California, USA; c Department of Biology, National and Kapodistrian University of Athens, Athens, Greece; d Los Angeles County + University of Southern California Medical Center, Los Angeles, California, USA; Baylor College of Medicine

**Keywords:** ESBL, *Escherichia coli*, One Health, antibiotic resistance, colicin, fiber, gut microbiome, host-pathogen interactions, infectious disease

## Abstract

Novel approaches to combating antibiotic resistance are needed given the ever-continuing rise of antibiotic resistance and the scarce discovery of new antibiotics. Little is known about the colonization dynamics and the role of intrinsic plant-food characteristics in this process. We sought to determine whether plant fiber could alter colonization dynamics by antibiotic-resistant bacteria in the gut. We determined that ingestion of antibiotics in mice markedly enhanced gut colonization by a pathogenic extended-spectrum beta-lactamase-producing Escherichia coli strain of human origin, E. coli JJ1886 (ST131-*H*30Rx). Furthermore, ingestion of soluble acacia fiber before and after antibiotic exposure significantly reduced pathogenic E. coli colonization. 16S rRNA analysis and *ex vivo* cocultures demonstrated that fiber protected the microbiome by serving as a prebiotic, which induced native gut E. coli to inhibit pathogenic E. coli via colicin M. Fiber may be a useful prebiotic with which to administer antibiotics to protect human and livestock gut microbiomes against colonization from antibiotic-resistant, pathogenic bacteria.

**IMPORTANCE** A One Health-based strategy—the concept that human health and animal health are interconnected with the environment—is necessary to determine the drivers of antibiotic resistance from food to the clinic. Moreover, humans can ingest antibiotic-resistant bacteria on food and asymptomatically, or “silently,” carry such bacteria in the gut long before they develop an opportunistic extraintestinal infection. Here, we determined that fiber-rich foods, in particular acacia fiber, may be a new, promising, and inexpensive prebiotic to administer with antibiotics to protect the mammalian (i.e., human and livestock) gut against such colonization by antibiotic-resistant, pathogenic bacteria.

## INTRODUCTION

With the continued rise in antibiotic resistance and diminishing discovery of new antibiotics, experts and public health organizations have warned about a postantibiotic era (1). It is critical to find ways to diminish societal exposure to antibiotics that drive selection for resistance (1, 2). The U.S. Centers for Disease Control and Prevention (CDC) has estimated that there are 2.8 million antibiotic-resistant infections and 35,000 resulting deaths per year in the United States (1, 3).

It has been well established that humans can asymptomatically carry antibiotic-resistant bacteria in the gut long before developing an opportunistic extraintestinal infection (4–8). Symptomatic patients presenting with acute extraintestinal infections only represent a fraction of humans serving as reservoirs for antibiotic-resistant bacteria and genes in the gut (9). One such study by Gurnee et al. identified antibiotic-resistant bacteria in more than 20% of stool from healthy human twins that had never been previously prescribed antibiotics (4). These findings stress the importance of identifying new solutions to prevent the gastrointestinal colonization and insidious proliferation of antibiotic-resistant bacteria to prevent further infections.

While the function of contaminated meat as a vehicle for the transmission of antibiotic-resistant bacteria from agriculture to the gut has been well described (2, 3, 10, 11), the role of plant-foods deserves more attention (11, 12). We recently demonstrated that bacterial colonization of plant-foods can result in the transmission of antibiotic resistance genes to and within the mammalian gut microbiome (13). However, we hypothesized that intrinsic plant characteristics could alter gut colonization dynamics. Dietary fiber is abundant in produce, and its benefits to gut health range from regulating bowel movements to promoting gut microbial ecology (14–16, 52). Fiber was also recently reported to have protective effects against ileal persistence of enterotoxigenic Escherichia coli (ETEC) in pigs (17). In this work, we have alternatively tested whether plant fiber could alter the colonization of extraintestinal pathogenic E. coli (ExPEC) in the host gut.

We focused on a Gram-negative, ExPEC clinical isolate harvested from blood and urine (JJ1886) (18). This strain is a member of the virulent ST131-*H*30Rx group, the most pervasive extended-spectrum beta-lactamase (ESBL) producers worldwide (9, 19, 20). The chromosomal *bla*_CTX-M-15_ ESBL gene results in resistance to all beta-lactam antibiotics except for the carbapenems (18). ESBL-producing bacteria are widespread in the environment, and they are typically carried in asymptomatic patients prior to extraintestinal infection (9, 19, 20). They are a rising cause of community onset infections and the only form of antibiotic-resistant bacteria that continues to increase in frequency across the United States. (21). As stewardship alone has been insufficient at slowing the spread of ESBL-producing E. coli (22), it is imperative to determine the drivers of its increasing colonization. Here, we developed a murine model in which mice were exposed to different antibiotics under various conditions to determine protective and antiprotective factors that altered pathogenic E. coli colonization persistence.

## RESULTS

### Asymptomatic E. coli colonization model.

**FIG 1:**
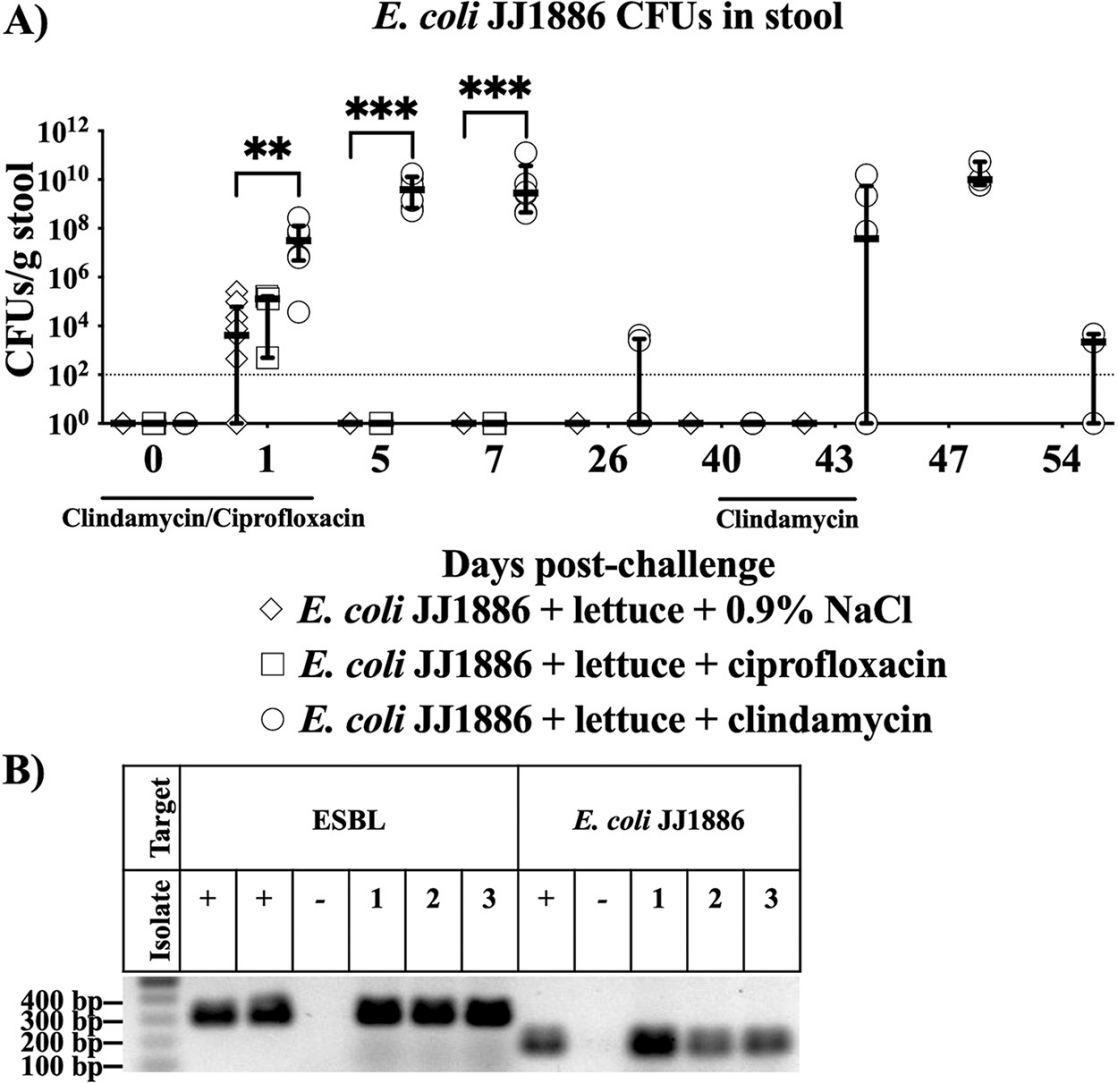
Clindamycin treatment resulted in stable mouse gut colonization by pathogenic E. coli JJ1886. (A) Mice were challenged with 10^7^ CFU E. coli JJ1886 with lettuce on day 0. Mice were treated with ciprofloxacin, clindamycin, or 0.9% NaCl saline placebo (*n* = 3, 6, or 9/group, respectively) from –2 to 1 day postchallenge and 40 to 43 days postchallenge for the clindamycin group. Data are medians ± interquartile range. Limit of detection (dotted line) = 10^2^ CFU/g stool. *, *P ≤ *0.05; **, *P ≤ *0.01; ***, *P ≤ *0.001; two-tailed Mann-Whitney test. (B) Confirmation of pathogenic E. coli JJ1886 persistence in the mouse gut after clindamycin selective pressure. Three CFU isolates (one per mouse with E. coli JJ1886 CFU) from stool collected 43 days postchallenge were selected for DNA confirmation. DNA was extracted, PCR amplified for ESBL and E. coli JJ1886 specific regions, and visualized. E. coli JJ1886 and Acinetobacter baylyi DNA were used as positive (+) and negative (–) controls, respectively.

To mimic ingestion of produce with pathogenic E. coli, we challenged mice with E. coli JJ1886 spiked into lettuce homogenate. Mice were inoculated with bacteria and lettuce during treatment with a 4-day course of ciprofloxacin, clindamycin, or saline placebo, mimicking patient exposure to a short course of antibiotics. Antibiotics were selected based on previously published anaerobic activity (19, 23). We successfully recovered E. coli JJ1886 from the stool on the day after infection (Fig. 1A), and colonization levels were significantly higher in clindamycin-treated mice (Fig. 1A). E. coli JJ1886 levels rapidly declined, such that we were unable to detect pathogenic E. coli JJ1886 by 5 days postchallenge in ciprofloxacin- and saline placebo-treated mice. In contrast, mice treated with clindamycin demonstrated significantly higher and longer gut colonization by E. coli JJ1886 (Fig. 1A). Nevertheless, by 26 days postchallenge, colonization had waned even in the presence of clindamycin.

However, we suspected that low-level E. coli JJ1886 colonization was persisting beyond 26 days postchallenge below the limit of detection of stool culture. Therefore, we applied a second course of selective pressure by administering clindamycin or saline placebo again starting from 40 days postchallenge (Fig. 1A). Repeat treatment with clindamycin allowed for the expansion of E. coli JJ1886 at 43 days postchallenge, confirming long-term colonization (Fig. 1A and B).

### Fiber curbed pathogenic gut colonization.

**FIG 2:**
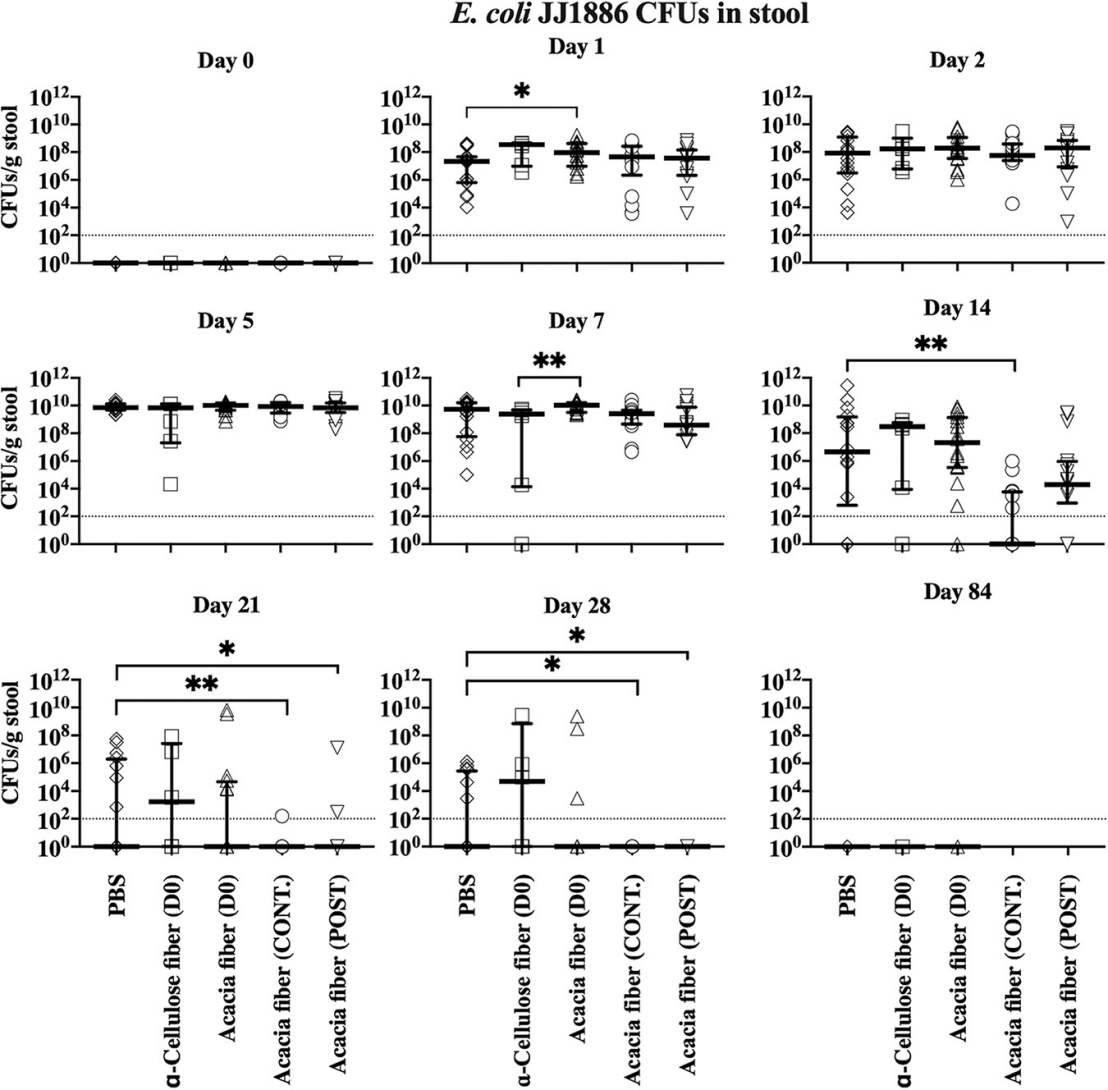
Soluble acacia fiber treatment decreased colonization by pathogenic E. coli JJ1886 in the murine gut despite clindamycin selective pressure. Mice were treated with clindamycin from –2 to 1 day postchallenge and challenged with 10^7^ CFU E. coli JJ1886 on day 0. Mice were given acacia fiber (soluble), α-cellulose fiber (insoluble), or PBS by oral gavage once at the time of infection (D0), for 18 days (CONT.), or for 4 days postchallenge (POST) (*n* = 16 per group except *n* = 6 for the α-cellulose fiber group). Data are medians ± interquartile range. Limit of detection (dotted line) = 10^2^ CFU/g stool. *, *P ≤ *0.05; **, *P ≤ *0.01; two-tailed Mann-Whitney test.

Using our ExPEC colonization model, we subsequently wanted to determine if fiber solubility altered pathogenic E. coli JJ1886 colonization. Soluble fibers are generally associated with an effect on gut microbial fermentation, whereas insoluble fibers are linked to mechanical influence of bowel movements (14). Lettuce is a source of insoluble cellulose fiber and other nutrients (24). To isolate the specific effect of fiber within the complex lettuce homogenate, the lettuce was replaced with insoluble α-cellulose. Additionally, acacia fiber was tested as an example of soluble fiber. Mice were treated with clindamycin from –2 to 1 days postchallenge in addition to a single dose of acacia fiber or α-cellulose on the day of infection. A single treatment with α-cellulose fiber (insoluble) resulted in greater inhibition of E. coli JJ1886 than a single treatment with acacia fiber (soluble) on day 7. However, there was no difference after 14 days postchallenge (Fig. 2). To minimize the confounding effect of insoluble fiber altering colonization simply by mechanically expediting microbial bowel transit, we continued to pursue soluble acacia fiber regimens.

To determine whether the dosing strategy of acacia fiber could be improved to further inhibit colonization by E. coli JJ1886, mice were again pretreated with clindamycin and treated with acacia fiber at either the time of infection (day 0), continuously pre- and postinfection for 18 days (–3 to 14 days postchallenge), or just postinfection (2 to 5 days postchallenge). Continuous treatment resulted in the most robust inhibition of E. coli JJ1886, with significantly fewer CFU recovered starting at 14 days postchallenge than for the phosphate-buffered saline (PBS) control (*P < *0.01, two-tailed Mann-Whitney test) (Fig. 2).

### Acacia fiber enhanced microbial antibiosis.

**FIG 3:**
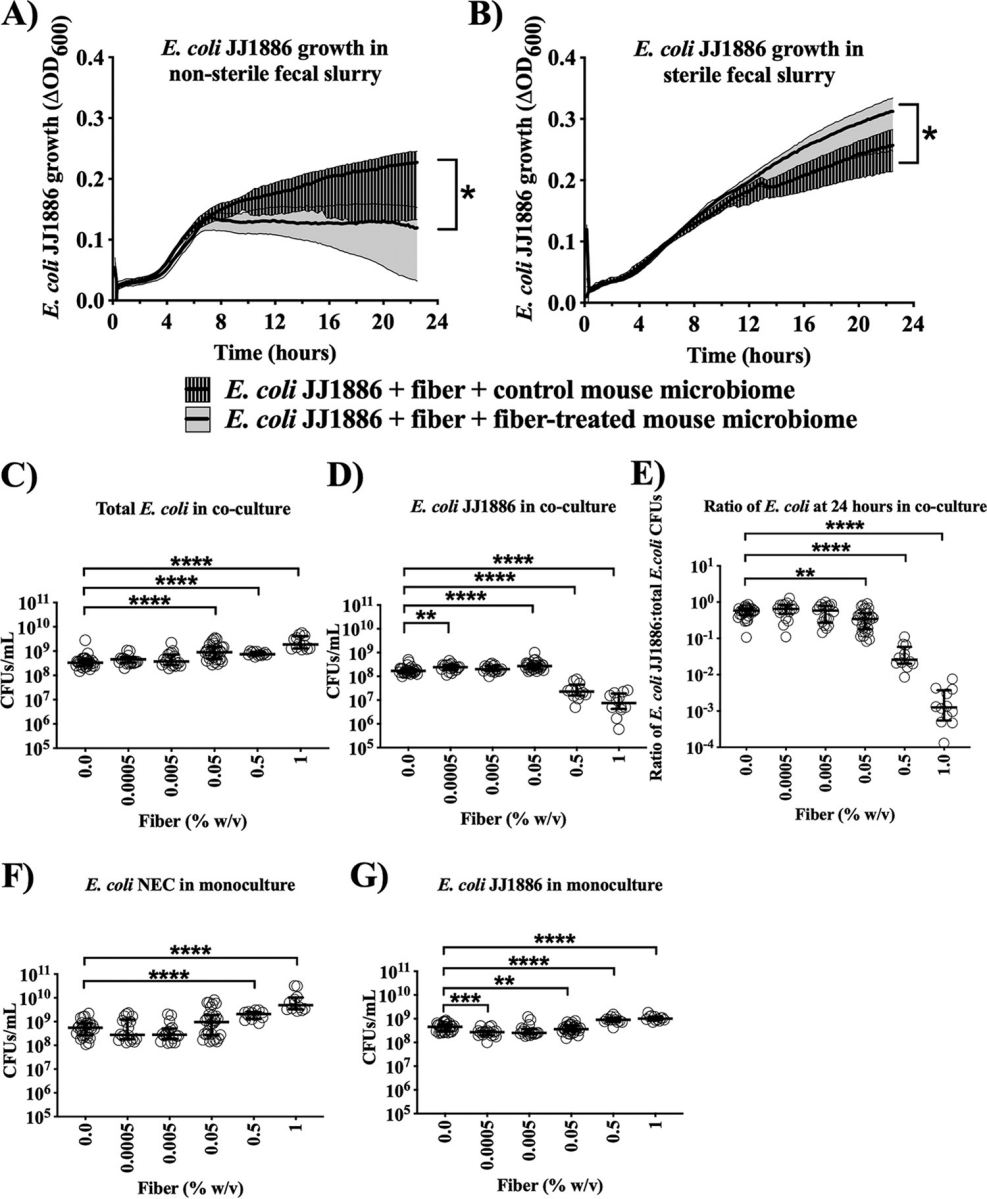
Pathogenic E. coli JJ1886 inhibition by native gut E. coli was acacia fiber dependent. (A and B) Fecal slurry created from stool of mice previously treated with fiber (fiber-treated mouse microbiome) and no fiber (control mouse microbiome) and (A) nonsterile or (B) filter-sterilized. Each well contained 100 μL of fecal slurry, 50 μL of 100 mg/mL fiber, and 50 μL of 10^6^ CFU E. coli JJ1886 (*n* = 8 per condition). Thick black lines = medians; shaded areas = interquartile range. (C to G) Native gut E. coli (NEC) isolates were combined from the stool of mice previously treated with fiber. E. coli NEC, E. coli JJ1886, or both were cultured with 0, 0.0005, 0.005, 0.05, 0.5, or 1% (wt/vol) fiber at a final concentration of 3 × 10^6^ CFU/mL. CFU were enumerated by plating on nonselective (C, F, and G) and selective (D) agar plates. (E) The ratio of E. coli JJ1886 to total E. coli in coculture was determined by dividing the selective CFU (D) by nonselective CFU (C). Data are medians ± interquartile range. *, *P ≤ *0.0001, nonlinear least-squares regression; **, *P ≤ *0.01; ***, *P ≤ *0.001; **** *P ≤ *0.0001; two-tailed Mann-Whitney test.

To investigate the microbiome-mediated effects of acacia fiber on E. coli JJ1886 persistence in the mouse gut, we established an *ex vivo* fecal slurry assay to determine whether the acacia fiber acted directly or indirectly on E. coli JJ1886 under aerobic conditions. Pathogenic E. coli JJ1886 was spiked into slurries made from feces taken from mice fed acacia fiber or the phosphate-buffered saline (PBS) control. Fecal slurry from fiber-treated mice, but not control mice, significantly suppressed E. coli JJ1886 density at 22.5 h postinoculation (*P < *0.0001, nonlinear least-squares regression) (Fig. 3A).

To determine if the fiber effect was direct upon the E. coli JJ1886 or required viable microbiota as an intermediary, we repeated the assay but filter-sterilized the fecal slurry before spiking in E. coli JJ1886. Filter sterilization abrogated suppression of E. coli JJ1886 growth in fecal slurry from fiber-treated mice (*P < *0.0001, nonlinear least-squares regression) (Fig. 3B). These results suggested that fiber-mediated suppression of E. coli JJ1886 growth required viable, native gut microbiota for a fiber-induced antibiosis effect, and hence acacia fiber worked via indirect changes to the microbiota as opposed to direct action against E. coli JJ1886.

### Acacia fiber enhanced colicin M-mediated inhibition.

**FIG 4:**
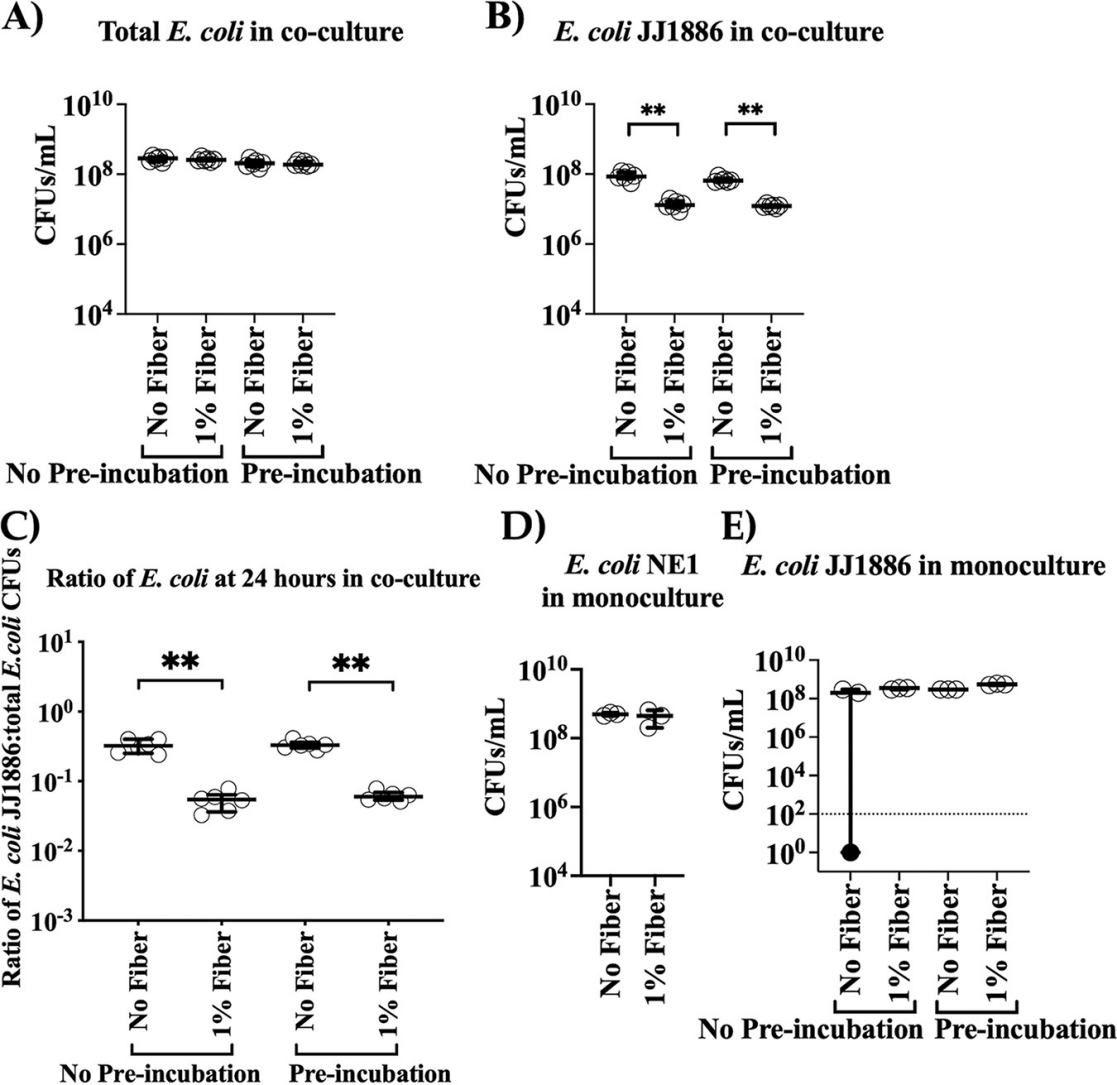
Preincubation of E. coli JJ1886 with fiber does not affect its inhibition by E. coli NE1. (A to E) E. coli NE1 and E. coli JJ1886 were grown in coculture (A to C) or monoculture (D and E) with ± 1% (wt/vol) acacia fiber. E. coli JJ1886 subculture was prepared with (Pre-incubation) or without (No Pre-incubation) 1% fiber for 2 h. Each well was prepared with 200 μL of M9 minimal medium ± 1% (wt/vol) acacia fiber and a final concentration of 3 × 10^6^ CFU/mL of E. coli JJ1886 or E. coli NE1 (*n* = 3 and 6 per monoculture and coculture, respectively). CFU were enumerated by plating on nonselective (A,D, and E) and selective (B) agar plates. Selective CFU (B) were divided by nonselective CFU (A) to determine the ratio of E. coli JJ1886 to total E. coli (C). (E) Limit of detection (dotted line) = 10^2^ CFU/g stool. Black circle denotes that the value was below the limit of detection. Data are medians ± interquartile range. **, *P ≤ *0.01; two-tailed Mann-Whitney test.

Given that clindamycin selects for *Enterobacteriaceae*, and E. coli are the predominant aerobic members of the gut microbiota and are known to mediate interstrain antibiosis (25–27), we hypothesized that fiber augmented endogenous gut E. coli antibiosis against E. coli JJ1886. We therefore selected from murine stool two native gut E. coli isolates (NE1 and NE3) 35 days post E. coli JJ1886 infection from mice treated with 18 days of daily acacia fiber (Fig. 2). We selected E. coli NE1 and NE3 specifically because they had persisted in the gut after antibiotic exposure, with fiber treatment, despite the hypothesized interstrain E. coli competition in the presence of E. coli JJ1886. Selective plating and matrix-assisted laser desorption ionization–time of flight mass spectrometry (MALDI-TOF) confirmed that the two isolates were wild-type, non-ESBL-producing E. coli, distinct from E. coli JJ1886. We conducted coculture experiments with the combined native E. coli isolates (NEC) and E. coli JJ1886. M9 minimal culture medium (pH 6.8) was used to mimic the nutrient-poor gut environment and the colonic luminal pH (28). Addition of acacia fiber to the coculture of E. coli NEC and E. coli JJ1886 significantly inhibited E. coli JJ1886 growth in a dose-dependent manner (Fig. 3C to E). However, adding fiber to monocultures of native E. coli or E. coli JJ1886 did not inhibit bacterial growth of either strain (Fig. 3F and G), reinforcing that the fiber acted indirectly through the native E. coli strains and not directly upon E. coli JJ1886. Furthermore, preincubation of E. coli JJ1886 with fiber before coculture with native E. coli also did not result in a growth difference of E. coli JJ1886 (Fig. 4).

**FIG 5:**
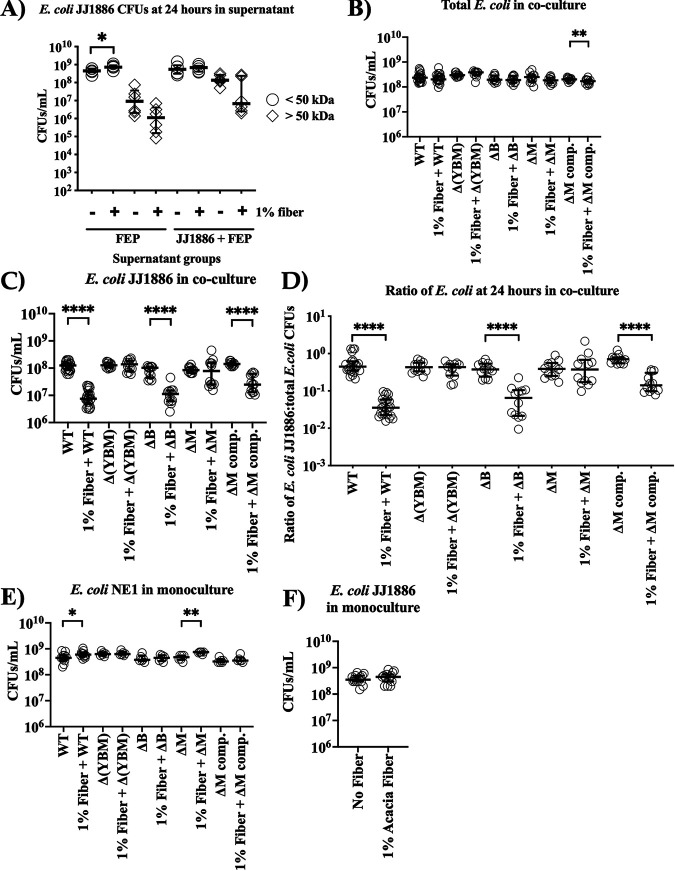
Native gut E. coli inhibited pathogenic E. coli JJ1886 with colicin M in the presence of acacia fiber. (A) E. coli NEC monoculture and coculture with E. coli JJ1886 were grown at a final concentration of 3 × 10^6^ CFU/mL with ± 1% (wt/vol) acacia fiber. Culture supernatant fractions smaller or larger than 50 kDa were incubated with E. coli JJ1886 at a final concentration of 3 × 10^6^ CFU/mL (*n* = 6 per condition). (B to F) E. coli NE1 ΔB, ΔM, and Δ(YBM) denote disrupted colicin activity genes *cba*, *cma*, or *cya*, *cba*, and *cma*, respectively. A final concentration of 3 × 10^6^ CFU/mL was used for E. coli JJ1886, E. coli NE1 wild type (WT) or mutant, or both. (B to D) E. coli JJ1886 was cocultured with ± 1% (wt/vol) acacia fiber and E. coli NE1 WT (*n* = 24), E. coli NE1 colicin knockout mutants (ΔYBM, ΔB, or ΔM) (*n* = 12), or E. coli NE1 ΔM complement (ΔM comp.) (*n* = 12). (D) CFU were enumerated by plating on nonselective (B) and selective (C) agar plates to determine the ratio of E. coli JJ1886:total E. coli. (E and F) E. coli monocultures with ± 1% (wt/vol) acacia fiber were performed in parallel as growth controls [*n* = 12, E. coli NE1 WT; *n* = 6, E. coli NE1 ΔB, ΔM, Δ(YBM)], and ΔM comp.; *n* = 15, E. coli JJ1886). Monoculture CFU were enumerated by plating on nonselective agar plates. Data are medians ± interquartile range. *, *P ≤ *0.05; **, *P ≤ *0.01; ****, *P ≤ *0.0001; two-tailed Mann-Whitney test.

Next, to determine if fiber induced native E. coli to secrete an extracellular product inhibiting E. coli JJ1886 growth, we size fractionated supernatants obtained from E. coli NEC monoculture and coculture with E. coli JJ1886 in the presence or absence of acacia fiber. We observed no inhibition of E. coli JJ1886 growth when it was incubated with supernatant fractions either larger or smaller than 50 kDa from native, fiber-exposed E. coli monoculture and coculture with E. coli JJ1886 (Fig. 5A).

These results suggested that fiber was acting via an inducible growth-inhibitory effect that required close and rapid cell-cell interaction, rather than being secreted/soluble. Colicins are defense proteins produced by E. coli to mediate antibiosis against other E. coli (27). Colicins can be membrane-bound, and therefore poorly soluble, or rapidly degraded by extracellular proteases (29, 30). We therefore screened the native E. coli for colicins. Sequencing confirmed colicins B and Y in both native E. coli isolates (NE1 and NE3) and additional colicin M in NE1 (Table S1). These colicins were absent in E. coli JJ1886.

10.1128/msphere.00071-22.2 TABLE S1 Sanger sequencing results. Download Table S1, DOCX file, 0.02 MB. Copyright © 2022 Maeusli et al. 2022 Maeusli et al. https://creativecommons.org/licenses/by/4.0/ This content is distributed under the terms of the Creative Commons Attribution 4.0 International license.

**FIG 6:**
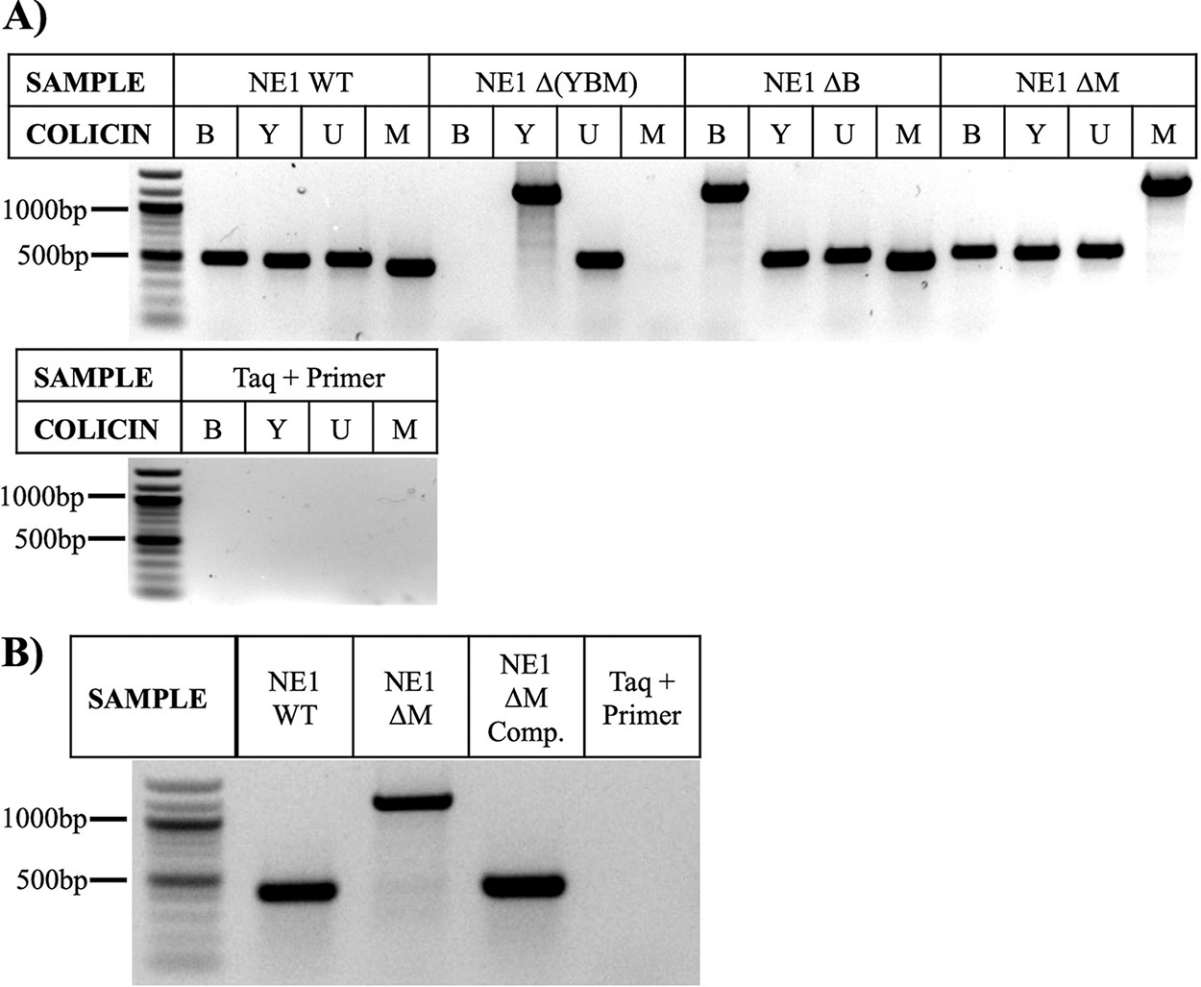
Confirmation of colicin knockout and complement E. coli NE1 strain construction. (A) A zeocin selective marker (776 bp) was inserted into the colicin activity genes harbored by E. coli NE1 wild-type (WT) bacteria. E. coli NE1 ΔB, ΔM, and Δ(YBM) denote disrupted colicin activity genes *cba*, *cma*, or *cya*, *cba*, and *cma*, respectively. Plasmid DNA was extracted, PCR amplified with primers targeting colicin activity genes, and visualized. Increased product size indicated successful insertion of the zeocin construct. Absent bands suggested off-target loss of the respective colicin activity gene. DNA sequencing confirmed nonspecific binding of published colicin U primers to colicin Y. E. coli NE1 WT served as the positive control. The *Taq* polymerase, primers, and water were run as a negative control. (B) E. coli NE1 ΔM comp. denotes the E. coli NE1 ΔM complement strain that was transformed with pTO4 containing colicin M activity (*cma*) and immunity (*cmi*) genes. Plasmid DNA was extracted, amplified, and visualized as described in panel A. Successful addition of *cma* into E. coli NE1 ΔM comp. resulted in a decreased product size equivalent to that of E. coli NE1 WT.

To establish whether colicins were involved in the fiber-mediated inhibition of E. coli JJ1886, we cocultured E. coli JJ1886 with E. coli NE1 or strains disrupted of colicin Y, B, or M (ΔY, ΔB, and ΔM respectively) in the presence or absence of acacia fiber. Disruption of colicin Y also resulted in the off-target disruption of colicin M and B as well [E. coli NE1 Δ(YBM)]. Abrogation of E. coli JJ1886 growth inhibition occurred only when the colicin M activity gene was disrupted (Fig. 5B to F and Fig. 6A). Complementation of the disrupted strain with the colicin M activity gene restored the growth-inhibitory effect of fiber during coculture, confirming that colicin M was the primary effector by which native E. coli inhibited ESBL-producing E. coli growth (Fig. 5B to F, 6B).

### Acacia fiber ameliorated antibiotic dysbiosis.

**FIG 7:**
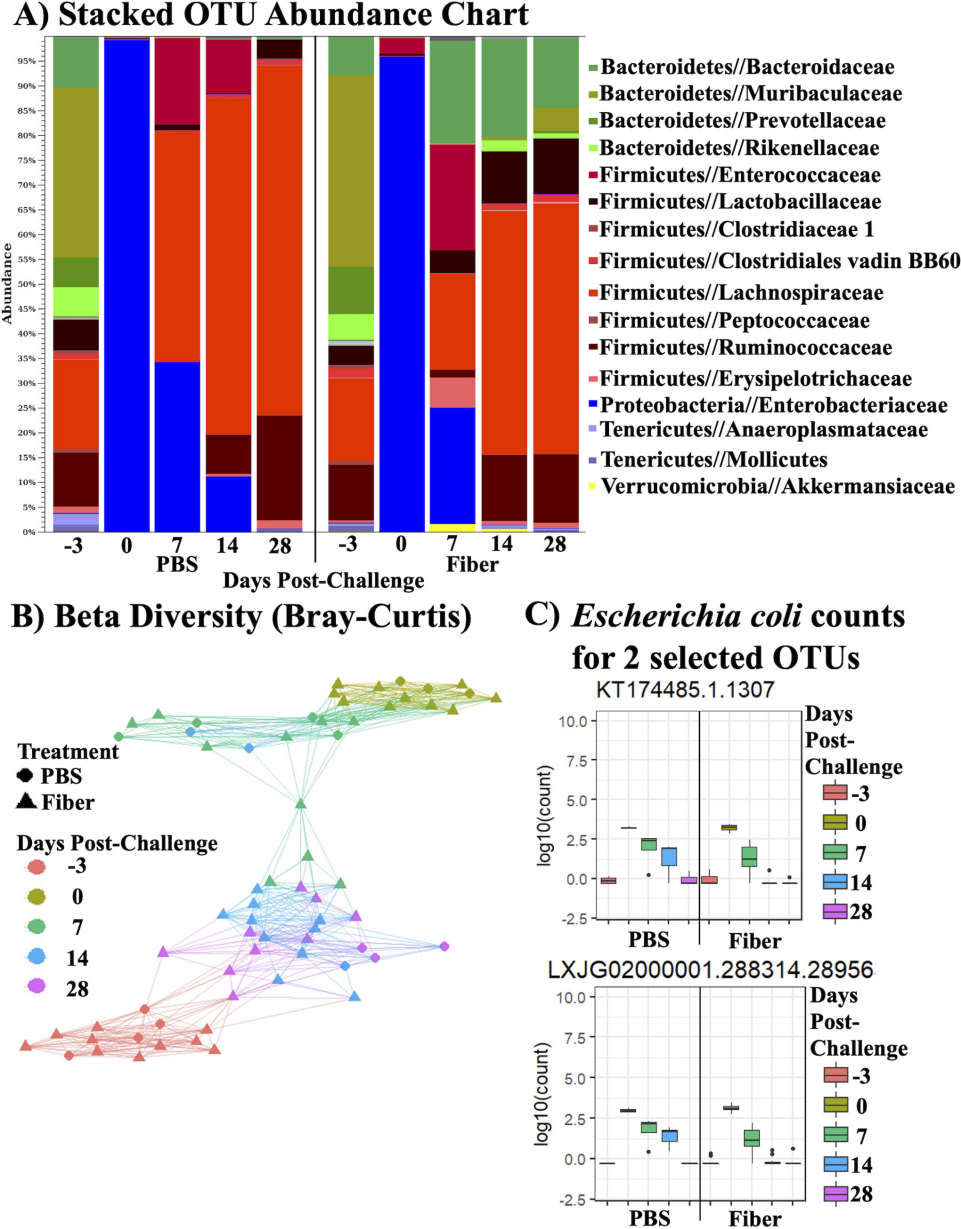
Acacia fiber ameliorated antibiotic dysbiosis in the gut by increasing microbial diversity. 16S rRNA Illumina sequencing of microbial DNA extracted from stool of mice in Fig. 2 treated with acacia fiber for 18 days (*n* = 10) or PBS (*n* = 4). Stool samples were collected at –3 (pretreatment, prechallenge), 0 (prechallenge), 7, 14, and 28 days (postchallenge). (A) Stacked bar chart of OTU abundance; legend depicts the 16 most abundant families. (B) Beta diversity similarity network as calculated by Bray-Curtis distances. (C) Abundance plots for select native gut E. coli OTUs (KT174485.1.1307 and LXJG02000001.288314.28956) showing OTU counts aggregated by treatment and day.

Lastly, to understand how the antibiotic and fiber were modulating colonization by ESBL-producing E. coli in the greater context of the anaerobic gut microbiome, we sought to define the gut microbiota under each condition. Microbial profiling of the fecal samples from continuous acacia fiber and PBS control-treated mice demonstrated that clindamycin had a dominant effect on altering the microbiota on the day of infection (i.e., after 3 days of continuous acacia fiber treatment, collected before E. coli JJ1886 infection) (Fig. 7A to C). Consistent with resistance of E. coli to clindamycin, operational taxonomic unit (OTU) abundances for native *Proteobacteria* (including E. coli) increased in both the PBS and fiber groups from –3 to 0 days postchallenge, whereas *Bacteroidetes* and *Firmicutes*, the major bacterial phyla in healthy guts (31), decreased (Fig. 7A).

We subsequently observed an increase in bacterial species abundance and evenness at 6 days after the last antibiotic treatment (i.e., 7 days postchallenge) in the fiber and PBS control groups, consistent with initial microbiome recovery (Fig. 7A and Fig. S1). However, recolonization of the gut differed in species richness between the fiber and PBS groups. Microbial dysbiosis due to clindamycin gradually shifted back to native conditions (i.e., pretreatment, prechallenge) by 28 days postchallenge (Fig. 7B). This shift in species richness was earlier in the fiber-treated murine stool, as indicated by the green and blue triangles representing 7- and 14-day postchallenge stool samples, respectively. Between 7 and 14 days postchallenge (after the end of antibiotic exposure), 133 OTUs of *Enterobacteriaceae* were reduced in fiber-treated mice, whereas no OTU shift was observed in the PBS control mice (Fig. 7A and Table S2). Closer analysis across the time points demonstrated that native E. coli isolates were inhibited in fiber-treated mice compared to those in PBS-treated mice. An example of this for 2 selected E. coli OTUs is presented in Fig. 7C. These results are consistent with fiber-induced E. coli antibiosis resulting from intensified strain-strain competition during recovery of the gut microbiome.

10.1128/msphere.00071-22.1 FIG S1 Alpha diversity (Shannon entropy) of fecal microbiota. 16S rRNA Illumina sequencing of microbial DNA extracted from stool of mice in Fig. 2 treated with acacia fiber for 18 days (*n* = 10) or PBS (*n* = 4). Stool samples were collected at –3 (pretreatment, prechallenge), 0 (prechallenge), 7, 14, and 28 days (postchallenge). No statistically significant differences were found between treatments by day (*P > *0.05, Wilcoxon rank sum test with Holm-Bonferroni adjustment). Download FIG S1, TIF file, 2.6 MB. Copyright © 2022 Maeusli et al. 2022 Maeusli et al. https://creativecommons.org/licenses/by/4.0/ This content is distributed under the terms of the Creative Commons Attribution 4.0 International license.

10.1128/msphere.00071-22.3 TABLE S2 Significant (*P < *0.05) OTU abundance contrasts between time points per bacterial family^a^.^*a*^Bacterial family OTU abundances were determined as described in **Fig. 7**. OTUs were identified per the SILVA reference database. Subsequently, abundance count data were processed for analysis in R (1). The DESeq2 package was implemented for the differential OTU analysis (2), in which OTU counts were modeled as the dependent variable in the context of a generalized linear model (GLM) with a negative binomial distribution, while treatment and day constituted the independent variables. Log2FC estimates with standard errors were estimated, and test statistics were performed. Comparisons were denoted in the following format: TREATMENT_DAYvsDAY. Treatments consisted of 18 days of acacia fiber (Fiber) or PBS control (PBS). Stool samples were collected at –3 (pretreatment, prechallenge), 0 (prechallenge), 7, 14, and 28 days (postchallenge). Download Table S2, DOCX file, 0.1 MB. Copyright © 2022 Maeusli et al. 2022 Maeusli et al. https://creativecommons.org/licenses/by/4.0/ This content is distributed under the terms of the Creative Commons Attribution 4.0 International license.

Furthermore, fiber treatment enhanced recolonization by other families between 7 and 14 days postchallenge, whereas recolonization was slower during recovery for those same families in placebo-treated mice. Specifically, 302 *Lachnospiraceae* and 78 *Ruminococcaceae* OTUs had significant fold increases in fiber-treated mice versus declines of 38 and 11 OTUs, respectively, in placebo mice (Table S2). Members of the *Bacteroidaceae* family were also responsive to fiber treatment. Thus, the fiber-induced expansion of non-*Enterobacteriaceae* families was concurrent with the inhibition of pathogenic E. coli JJ1886 growth through 14 and 28 days postchallenge.

## DISCUSSION

We explored the persistence of antibiotic-resistant bacteria consumed with plant-foods in an asymptomatic colonization model and defined factors that protect against pathogenic, ESBL-producing E. coli colonization of the native gut. We found that the native murine gut is relatively resistant to ESBL-producing E. coli colonization, even at high bacterial inocula. However, the dysbiotic effects of clindamycin potently enabled colonization. These results mimic the exposure of humans and livestock to short courses of antibiotics, likely rendering them more susceptible to colonization by antibiotic-resistant bacteria to which they are regularly exposed on meat and produce (11, 12, 32–34). Of great importance, continuous acacia fiber treatment inhibited pathogenic E. coli colonization, counteracting the effects of clindamycin on the native gut microbiome, which is in congruence with a recent finding that diet affects E. coli persistence in the gut (35).

Fiber enhanced recovery of a diverse microbial ecology in the native gut, which appeared to force strain competition that resulted in intraspecies E. coli antibiosis, whereas in placebo-treated mice the less robust ecological recovery did not force such strain competition, despite clindamycin’s selective pressure favoring *Enterobacteriaceae*. These results were consistent with the fiber-induced expansion of families other than *Enterobacteriaceae* families, forcing interstrain E. coli competition that inhibited E. coli JJ1886 growth. The fiber-enhanced antibiosis effect inhibiting the ESBL-producing E. coli required and was mediated by colicin M, a membrane-bound and poorly soluble colicin (29, 36), in the native E. coli strains. An improved understanding of acacia fiber interaction with native E. coli at the cellular level for colicin M induction may identify new protective targets against colonization by antibiotic-resistant bacteria.

In summary, we have confirmed that ingestion of acacia fiber ameliorated antibiotic-induced colonization of the gut by a pathogenic, clinical isolate of ESBL-producing E. coli. Fiber enabled more rapid recovery of host microbiota after antibiotic exposure and stimulated expression of colicin M among native E. coli to inhibit growth of the invading pathogenic, antibiotic-resistant strain. Given the 11.5 million kg of antimicrobials sold for use in food animals (excluding crop production) and the approximately 258.2 million patients prescribed oral antibiotics in the United States alone, fiber may be a promising, novel, safe, and inexpensive prebiotic to administer as an adjunct to antibiotic therapy for patients and in agricultural settings (e.g., livestock) to protect native gut microbiomes from colonization by antibiotic-resistant, pathogenic strains (37, 38). Additional work is needed to further characterize fiber types and doses to optimize the effect and to identify other food characteristics that can exacerbate or ameliorate antibiotic-resistant bacterial colonization of the mammalian gut.

## MATERIALS AND METHODS

### Plant growth.

Buttercrunch lettuce (Lactuca sativa, Reimer Seeds) was chosen as the plant material for the model because leafy vegetables are susceptible to cross-contamination and consumed raw, and lettuce is a dominant global commodity (12, 39, 40). We grew the lettuce in a growth chamber to avoid the high quantities of colonizing bacteria on purchased lettuce (41). The lettuce was grown under environmentally controlled conditions in a Panasonic MLR-352-PA plant growth chamber as previously published (13, 42).

### Mouse drug treatment.

Female, Murine Pathogen Free BALB/c mice aged 8 to 9 weeks were obtained from Taconic Biosciences. A total of 2 or 3 mice per cage were housed together. Mice in antibiotic-treated groups were given 200 μL of 30 mg/kg of body weight ciprofloxacin, 100 mg/kg clindamycin, or 0.9% NaCl saline placebo via subcutaneous injection once daily for 4 days (–2 to 1 day postchallenge). Mice treated with secondary clindamycin treatment were treated for 3 additional days (40 to 43 days postchallenge).

### Mouse oral gavage.

The E. coli JJ1886 inoculum was prepared from a frozen stock (43) to a concentration of 10^8^ CFU/mL in PBS. The inoculum was verified immediately before and after mouse infection by performing serial dilutions and plating in triplicate on tryptic soy agar (TSA) plates. TSA plates were incubated at 37°C for 24 h before CFU enumeration.

We administered the infectious inocula and oral treatments using a 1-mL syringe attached to a polypropylene oral gavage needle (20 gauge (ga) by 38 mm). Gavage was performed on all mice with 10^7^ CFU E. coli JJ1886 in 100 μL of PBS control, with or without 0.33 g/mL lettuce homogenate, 100 mg/mL soluble acacia fiber (RenewLife, 100% acacia fiber, Acacia senegal), or 100 mg/mL insoluble α-cellulose fiber (Sigma-Aldrich) as appropriate on day 0 (D0; 0 days postchallenge). Additional 100-μL soluble and insoluble fiber treatments were given as appropriate by oral gavage daily for 18 days (CONT.; –3 to 14 days postchallenge) or only postinfection (POST; daily, 2 to 5 days postchallenge). An Omni tissue homogenizer was used to make the lettuce homogenate in PBS.

### Fecal sample collection.

Two stool pellets per mouse were collected at each time point, one for CFU quantification and another for genomic DNA (gDNA) fecal extraction. Fecal samples were collected between 8 and 10 a.m. as published by others (44). Stool samples were collected on –3 or –2 days postchallenge as appropriate as pretreatment, prechallenge controls. All fecal samples collected at 0 days postchallenge were collected immediately before infection. Postinfection samples were collected 1, 2, 5, and 7 days postchallenge and weekly thereafter unless noted otherwise. Fecal samples for gDNA extraction were stored at –20°C until ready for use.

Fecal pellets for CFU quantification were weighed and resuspended in 1 mL PBS. Each microcentrifuge tube containing a fecal sample was then vortexed at maximum speed for 10 min to homogenize the fecal pellet. The fecal homogenate samples were then serially diluted using the drop plate method (45) and plated on eosin methylene blue medium (EMB) with 32 μg/mL cefepime and 100 μg/mL ampicillin (AMP) for selection of antibiotic-resistant colonies. CFU were then counted after overnight incubation at 37°C without shaking.

### Genomic extraction and confirmation.

Total DNA was extracted from fecal pellets using the Quick-DNA fecal/soil microbe kit (Zymo Research) per the manufacturer’s protocol, with a modification: DNA/RNA Shield (Zymo Research) was used in place of BashingBead buffer (Zymo Research) at the bead beating step to ensure DNA stability. Plasmid DNA was isolated from E. coli using commercial kits (GeneJET, Thermo Fisher). DNA was quantified using the Take3 microvolume plate (BioTek). PCR was performed using the primers listed in Table S3. PCR products were visualized on agarose gels and sequenced by Sanger sequencing (Table S1).

10.1128/msphere.00071-22.4 TABLE S3 Summary of primers. Download Table S3, DOCX file, 0.02 MB. Copyright © 2022 Maeusli et al. 2022 Maeusli et al. https://creativecommons.org/licenses/by/4.0/ This content is distributed under the terms of the Creative Commons Attribution 4.0 International license.

### Fecal slurry assay.

Stool samples were collected at 0 days postchallenge (Fig. 2) from mice that were treated with acacia fiber once daily for 4 days (i.e., –3 to 0 days postchallenge) or no fiber, as previously described, to create fecal slurry adapted from Nagpal et al. representative of the aerobic gut microbiota (46). Stool pellets were pooled by treatment, resuspended to 10 mg/mL in PBS, and homogenized for 10 min by vortexing at maximum speed. Homogenates were centrifuged at 35 × *g* for 1 min to remove large particulate matter, resulting in the nonsterile fecal slurry. Sterile fecal slurry was made by passing the nonsterile slurry through a 0.22-μm filter. E. coli JJ1886 was prepared from frozen stock as previously described. Using a 96-well plate, each well was filled with 100 μL fecal slurry (sterile or nonsterile) from fiber-treated or PBS control mice, 0.5 mg soluble acacia fiber, and 5 × 10^4^ CFU E. coli JJ1886. Control wells consisted of fecal slurry (sterile or nonsterile) and acacia fiber without E. coli JJ1886. Each well condition was performed in 8 replicates. The plate was incubated at 37°C with shaking on a plate reader. Optical density at 600 nm (OD_600_) measurements were recorded every 10 min for 22.5 h to estimate bacterial density. The median OD_600_ reading from the slurry control wells were used to normalize E. coli JJ1886 growth in each well.

### *Ex vivo* E. coli coculture with fiber.

Native gut E. coli was collected from fecal samples of 4 mice treated with 18 days of daily acacia fiber. At 35 days postchallenge, 1 fecal pellet per mouse was collected, and fecal pellets from all mice were pooled, resuspended in PBS, plated on nonselective eosin-methylene blue (EMB) plates, and incubated overnight as previously described. To ensure exclusion of remnant antibiotic-resistant E. coli JJ1886, nonselective plates were duplicated by velvet stamps on double selective EMB plates and incubated overnight at 37°C. Meanwhile, nonselective plates were stored at 4°C overnight. After confirming no growth on selective plates, two native gut E. coli colonies (native Escherichia coli sample 1, or NE1, and NE3) were isolated from the nonselective plates and confirmed using MALDI-TOF. E. coli NE1 and E. coli NE3 were combined (NEC) for initial *ex vivo* coculture experiments with E. coli JJ1886. All E. coli strains summarized in Table S4 were grown in overnight broth cultures with 10 mL tryptic soy broth (TSB) at 37°C/200 rpm. Subcultures were made with 100 μL of the respective E. coli overnight cultures in 10 mL TSB or TSB with 1% (wt/vol) acacia fiber as appropriate at 37°C/200 rpm for 2 h. Subcultures were centrifuged at 3,488 × *g*/5 min, resuspended in 10 mL PBS, and washed 3 times. Bacterial cultures were adjusted to an OD_600_ of 0.5.

10.1128/msphere.00071-22.5 TABLE S4 Summary of bacterial strains and plasmids used in this study. Download Table S4, DOCX file, 0.02 MB. Copyright © 2022 Maeusli et al. 2022 Maeusli et al. https://creativecommons.org/licenses/by/4.0/ This content is distributed under the terms of the Creative Commons Attribution 4.0 International license.

Using a 96-well plate, monocultures and cocultures of E. coli JJ1886 and native gut E. coli (NE1 or NEC as appropriate) were grown for 24 h with or without shaking at 37°C/200 rpm. Monocultures were prepared to a final concentration of 3 × 10^6^ CFU/mL of JJ1886 or native gut E. coli in 200 μL of M9 minimal medium (BD Biosciences). Cocultures consisted of 3 × 10^6^ CFU/mL of each E. coli strain (JJ1886 and either NE1 or NEC). M9 minimal medium was made per the manufacturer’s protocol except that glucose was adjusted to a final concentration of 0.05% (wt/vol) to minimize glucose as a carbon source. Acacia fiber was added to the M9 medium at 0.0005%, 0.005%, 0.01% 0.05%, 0.1%, 0.5%, or 1% (wt/vol) as appropriate and sterilized through a 0.22-μm filter. Each well condition was performed in 3, 6, 12, 18, or 30 replicates as appropriate.

At the 24-h time point, 20 μL was removed from each well and transferred to a new 96-well plate for serial dilution and drop plating (45) in duplicate on selective and nonselective EMB plates. Plates were incubated overnight at 37°C without shaking. Total E. coli and E. coli JJ1886 CFU were quantified from nonselective and selective plates, respectively. The E. coli JJ1886 CFU (selective) were divided by the total E. coli CFU (nonselective) to obtain the ratio of E. coli JJ1886 to total E. coli.

### Bacterial culture supernatant assay.

E. coli NEC monocultures and cocultures with E. coli JJ1886 were grown with 0 or 1% (wt/vol) acacia fiber in 15 mL of M9 minimal medium at a final concentration of 3 × 10^6^ CFU/mL at 37°C/200 rpm for 24 h. M9 minimal medium was prepared as previously described. Cultures were pelleted by centrifugation at 18 kG/10 min. Next, 4 mL of each culture supernatant was then size fractionated through a 50-kDa centrifugal spin column (Amicon) at 4,000 rpm/15 min. Supernatant fractions consisting of molecules larger than 50 kDa were resuspended in PBS to adjust to the prefractionation volume. Then, 2 mL of supernatant fractions either smaller or larger than 50 kDa were incubated with 2 mL of M9 minimal medium with 0 or 1% (wt/vol) acacia fiber as appropriate and E. coli JJ1886 at a final concentration of 3 × 10^6^ CFU/mL at 37°C/200 rpm for 24 h. E. coli JJ1886 CFU were enumerated by plating on selective EMB agar as described above.

### Colicin knockout and complement strains.

Using the nucleotide sequences for each colicin, knockout fragments were designed by insertion of a zeocin selective marker into colicin B, Y, U, and M activity genes (*cba*, *cya*, *cua*, and *cma*, respectively). E. coli NE1::pKM200 was used for lambda red-mediated homologous recombination (47) to insert the zeocin construct (Table S5) (48). Additionally, colicin M was restored to the E. coli NE1 colicin M knockout mutant (NE1 ΔM) for construction of the complemented strain (NE1 ΔM comp.). This was done by electrotransformation with plasmid pTO4 and mutant selection on TSA with 100 μg/mL AMP. Colicin knockout and complemented E. coli NE1 strains were cocultured with E. coli JJ1886 in the presence or absence of fiber as described above.

10.1128/msphere.00071-22.6 TABLE S5 Colicin knockout E. coli mutant DNA sequences with zeocin insertion. Download Table S5, DOCX file, 0.02 MB. Copyright © 2022 Maeusli et al. 2022 Maeusli et al. https://creativecommons.org/licenses/by/4.0/ This content is distributed under the terms of the Creative Commons Attribution 4.0 International license.

### *Ex vivo* and *in vivo* statistical analyses.

Statistical tests were performed using GraphPad Prism except for the case of the gut microbial profiling described below. Mann-Whitney and Kruskal-Wallis with Dunn’s multiple-comparison tests were used for nonparametric comparison of two and multiple groups, respectively. Nonlinear least-squares regression was performed for fecal slurry assays. *P* values of ≤0.05 were considered significant.

### Gut microbiota profiling.

Genomic DNA samples were extracted from 70 murine fecal samples using the aforementioned methods. Library preparation and next-generation sequencing were performed at the Children’s Hospital Los Angeles Single Cell, Sequencing, and CyTOF (SC2) Core Laboratory. 16S rRNA sequencing of the V4 region was performed on an Illumina MiSeqDx instrument with an Illumina MiSeq sequencing reagent kit v2 (300 cycles; 15 million reads).

Operational taxonomic units (OTUs) were assembled and matched against the SILVA database (49) to generate abundance tables using the microbial module of CLC Genomics Workbench 20 software (Qiagen). Stacked bar charts were produced using the same tool. Microbiome OTU data analysis was performed using the phyloseq package in R (50). Alpha diversity was expressed by Shannon’s index for each treatment group and time point. Statistically significant differences on Shannon’s index estimates by time point were displayed using the Wilcoxon ranked sum test. Microbiome differentiation among samples was quantified by Bray-Curtis distance measurements using the 2,000 most abundant OTUs. The differential OTU abundance analysis was executed within the DESeq2 package (51). OTU counts were modeled using the negative binomial distribution, and comparison contrasts were formulated by combinations of treatment and time point. Adjusted *P* values were estimated by the Benjamini-Hochberg method, and comparisons were considered significant when the adjusted (adj.) *P* value was <0.05. Shrunken log_2_ fold changes were calculated using the function lfcShrink.

### Study approval.

All animal work was done with the approval of the Institutional Animal Care and Use Committee at the University of Southern California’s Keck School of Medicine (IACUC 20837).

### Data and materials availability.

The sequencing data have been deposited in the National Center for Biotechnology Information Sequence Read Archive (BioProject number PRJNA758488). Correspondence and requests for materials should be addressed to the corresponding authors.
