# Supplementary material for: Acacia Fiber Protects the Gut from Extended-Spectrum Beta-Lactamase (ESBL)-Producing Escherichia coli Colonization Enabled by Antibiotics
Source: mSphere. 2022 May 18;7(3):e00071-22. doi: 10.1128/msphere.00071-22 (PMC9241499; doi:10.1128/msphere.00071-22)
Supplement: TABLE S1 [file msphere.00071-22-s0001.docx]

| **Table S1: Sanger Sequencing Results** | | | |
| --- | --- | --- | --- |
| ***E. coli* Isolate** | **Target Colicin** | **Primer** | **Sequencing Product** |
| NE1 | B | LEE0097 | ATCATTAGTGTCGGTGACAAAGTCGGGGAATATCTTGGAGATAAATACAAGGCGCTTTCCCGTGAAATTGCAGAGAATATAAATAATTTTCAGGGAAAAACGATTCGTAGTTATGATGATGCAATGTCTTCCATTAATAAGTTAATGGCTAACCCCAGCCTTAAAATAAATGCAACGGACAAAGAAGCCATTGTGAATGCGTGGAAAGCATTTAATGCTGAGGATATGGGGAATAAATTTGCTGCGTTGGGTAAAACGTTCAAAGCAGCAGATTATGCAATAAAGGCAAACAACATCAGGGAGAAGAGTATTGAGGGTTACCAGACTGGTAACTGGGGGCCATTAATGCTGGAAGTCGAGTCCTGGGTTATCAGTGGGATGGCATCTGCTGTAGCTCTTAGTTTGTTTTCTTTGACATTAGGCTCGGCCCTTATAGCCT |
| NE1 | B | LEE0098 | AGAGCTACAGCAGATGCCATCCCACTGATAACCCAGGACTCGACTTCCAGCATTAATGGCCCCCAGTTACCAGTCTGGTAACCCTCAATACTCTTCTCCCTGATGTTGTTTGCCTTTATTGCATAATCTGCTGCTTTGAACGTTTTACCCAACGCAGCAAATTTATTCCCCATATCCTCAGCATTAAATGCTTTCCACGCATTCACAATGGCTTCTTTGTCCGTTGCATTTATTTTAAGGCTGGGGTTAGCCATTAACTTATTAATGGAAGACATTGCATCATCATAACTACGAATCGTTTTTCCCTGAAAATTATTTATATTCTCTGCAATTTCACGGGAAAGCGCCTTGTATTTATCTCCAAGATATTCCCCGACTTTGTCACCGACACTAATGATGACCTCGCTGGTTTTTGTCAGGACCGTCT |
| NE1 | Y | LEE0135 | AGCTGAAGCTGAAAAAGCGGCTGCTGAAGCAAAAGCAAAAGCTGAAGCTGAAAAAGCCAGAAAGGAAGCTGAAGCAAAAGCAAATAACGAGAAAGCTGTTCTGACAAAAGCCAGTGAAATTATCATTAGTGTGGGTGATAAGGTCGGAGAATATCTTGGCGATAAATATAAAGCTCTTTCTCGTGAGATAGCAGGTAATATCAAAAACTTTCAAGGTAAGACGATCCGTAGTTATGATGAGGCGATGGCTTCTGTCAATAAACTGATGGCTAATCCTGATCTTAAAATTAATGCTGCAGACAGGGATGTCATTGTGAATGCCTGGAAAGCATTTGATGCAGAGGATATGGGGAATAAGTTTGCCGCGCTGGGTAAGACATTTAAAGCCGCAGATTATGTGATGAAGGCAA |
| NE1 | Y | LEE0136 | ATTCCCCATATCCTCTGCATCAAATGCTTTCCAGGCATTCACAATGACATCCCTGTCTGCAGCATTAATTTTAAGATCAGGATTAGCCATCAGTTTATTGACAGAAGCCATCGCCTCATCATAACTACGGATCGTCTTACCTTGAAAGTTTTTGATATTACCTGCTATCTCACGAGAAAGAGCTTTATATTTATCGCCAAGATATTCTCCGACCTTATCACCCACACTAATGATAATTTCACTGGCTTTTGTCAGAACAGCTTTCTCGTTATTTGCTTTTGCTTCAGCTTCCTTTCTGGCTTTTTCAGCTTCAGCTTTTGCTTTTGCTTCAGCAGCCGCTTTTTCAGCTTCAGCTTTCGCCTTTGCATCAGCTTTTGCTTTTTCTGCTGCCGCCTTGTTCTT |
| NE1 | U | LEE0133 | CTCCTTCTGGTGTTATGCAGGTCACCATTGAGGGGATAACCTCCACTCAGGCCAAGCAGCTTGGTTTGGGAGGGCTGGTCATGGGATATAACGCTTCTGGTGTTATTGGGGCTGTGGGCGAAATAGACACCGGTCACCGCCTTAATGCATCAGGAGCAAGTACTCCCGGAAGCGAAACTTCTGTGGAGAGTTTTGTGAACGGACAGAAACCCGCCGGAGAATGGCATGCTGTGGCGAAGGACAGCTGGACAGGGGCTGGTCCAGTGAATGTAGGGCTGGTTAATAACGCCATCAAAAGTGTCCGGATTATCAAAAAAGGCTATGTGACTGGTGTTCTCTTGCCTGAAGAGGTGATGAATAAAGCAGAGTACAAAGCAATGCGGCAGGCTTTCGATTCCCTTCCACTGGCAAAAC |
| NE1 | U | LEE0134 | CATTGCTTTGTACTCTGCTTTATTCATCACCTCTTCAGGCAAGAGAACACCAGTCACATAGCCTTTTTTGATAATCCGGACACTTTTGATGGCGTTATTAACCAGCCCTACATTCACTGGACCAGCCCCTGTCCAGCTGTCCTTCGCCACAGCATGCCATTCTCCGGCGGGTTTCTGTCCGTTCACAAAACTCTCCACAGAAGTTTCGCTTCCGGGAGTACTTGCTCCTGATGCATTAAGGCGGTGACCGGTGTCTATTTCGCCCACAGCCCCAATAACACCAGAAGCGTTATATCCCATGACCAGCCCTCCCAAACCAAGCTGCTTGGCCTGAGTGGAGGTTATCCCCTCAATGGTGACCTGCATAACACCAGAAGGAGCAATACTTCCCAGAATCATTTTTGCTTCAGGATTCATTTTT |
| NE1 | M | LEE0127 | CATGAATATGAAACAAATGAGCGGTAATGTCACTACACCAATTGTGGCGCTTGCTCACTATTTATGGGGTAATGGCGCTGAAAGGAGCGTTAATATCGCCAACATTGGTCTTAAAATTTCCCCTATGAAAATTAATCAGATAAAAGACATTATAAAATCTGGTGTAGTAGGTACATTCCCTGTTTCTACAAAGTTCACACATGCCACTGGTGATTATAATGTTATTACCGGTGCATATCTTGGTAATATCACACTGAAAACAGAAGGTACTTTAACTATCTCTGCCAATGGCTCCTGGACTTACAATGGCGTTGTTCGTTCATATGATGATAAATACGATTTTAACGCCAGCACTCACCGTGGCATT |
| NE1 | M | LEE0128 | ACGAACAACGCCATTGTAAGTCCAGGAGCCATTGGCAGAGATAGTTAAAGTACCTTCTGTTTTCAGTGTGATATTACCAAGATATGCACCGGTAATAACATTATAATCACCAGTGGCATGTGTGAACTTTGTAGAAACAGGGAATGTACCTACTACACCAGATTTTATAATGTCTTTTATCTGATTAATTTTCATAGGGGAAATTTTAAGACCAATGTTGGCGATATTAACGCTCCTTTCAGCGCCATTACCCCATAAATAGTGAGCAAGCGCCACAATTGGTGTAGTGACATTACCGCTCATTTGTTTCATATTCATTGAGCGATAGTCATATTGACTTGGCGCTGGTTTTG |
| NE3 | B | LEE0097 | ACAAAGTCGGGGAATATCTTGGAGATAAATACAAGGCGCTTTCCCGTGAAATTGCAGAGAATATAAATAATTTTCAGGGAAAAACGATTCGTAGTTATGATGATGCAATGTCTTCCATTAATAAGTTAATGGCTAACCCCAGCCTTAAAATAAATGCAACGGACAAAGAAGCCATTGTGAATGCGTGGAAAGCATTTAATGCTGAGGATATGGGGAATAAATTTGCTGCGTTGGGTAAAACGTTCAAAGCAGCAGATTATGCAATAAAGGCAAACAACATCAGGGAGAAGAGTATTGAGGGTTACCAGACTGGTAACTGGGGGCCATTAATGCTGGAAGTCGAGTCCTGGGTTATCAGTGGGATGGCATCTGCTGTAGCTCTTAGTTTGTTTTCTTTGACATTAGGCTCGGCCCTTATAG |
| NE3 | B | LEE0098 | GATGCCATCCCACTGATAACCCAGGACTCGACTTCCAGCATTAATGGCCCCCAGTTACCAGTCTGGTAACCCTCAATACTCTTCTCCCTGATGTTGTTTGCCTTTATTGCATAATCTGCTGCTTTGAACGTTTTACCCAACGCAGCAAATTTATTCCCCATATCCTCAGCATTAAATGCTTTCCACGCATTCACAATGGCTTCTTTGTCCGTTGCATTTATTTTAAGGCTGGGGTTAGCCATTAACTTATTAATGGAAGACATTGCATCATCATAACTACGAATCGTTTTTCCCTGAAAATTATTTATATTCTCTGCAATTTCACGGGAAAGCGCCTTGTATTTATCTCCAAGATATTCCCCGACTTTGTCACCGACACTAATGATGACCTCGCTGGTTTTTGTCAGGACCGTCTT |
| NE3 | Y | LEE0135 | TGCAAGGCGAAGCTGAAGCTGAAAAAGCGGCTGCTGAAGCAAAAGCAAAAGCTGAAGCTGAAAAAGCCAGAAAGGAAGCTGAAGCAAAAGCAAATAACGAGAAAGCTGTTCTGACAAAAGCCAGTGAAATTATCATTAGTGTGGGTGATAAGGTCGGAGAATATCTTGGCGATAAATATAAAGCTCTTTCTCGTGAGATAGCAGGTAATATCAAAAACTTTCAAGGTAAGACGATCCGTAGTTATGATGAGGCGATGGCTTCTGTCAATAAACTGATGGCTAATCCTGATCTTAAAATTAATGCTGCAGACAGGGATGTCATTGTGAATGCCTGGAAAGCATTTGATGCAGAGGATATGGGGAATAAGTTTGCCGCGCTGGGTAAGACATTTAAAGCCGCAGATTATGTGATGAAG |
| NE3 | Y | LEE0136 | GCGCGGCAAACTTATTCCCCATATCCTCTGCATCAAATGCTTTCCAGGCATTCACAATGACATCCCTGTCTGCAGCATTAATTTTAAGATCAGGATTAGCCATCAGTTTATTGACAGAAGCCATCGCCTCATCATAACTACGGATCGTCTTACCTTGAAAGTTTTTGATATTACCTGCTATCTCACGAGAAAGAGCTTTATATTTATCGCCAAGATATTCTCCGACCTTATCACCCACACTAATGATAATTTCACTGGCTTTTGTCAGAACAGCTTTCTCGTTATTTGCTTTTGCTTCAGCTTCCTTTCTGGCTTTTTCAGCTTCAGCTTTTGCTTTTGCTTCAGCAGCCGCTTTTTCAGCTTCAGCTTTCGCCTTTGCATCAGCTTTTGCTTTTTCTGCTGCCGCCTTGTTCT |
| NE3 | U | LEE0133 | ATTGCTCCTTCTGGTGTTATGCAGGTCACCATTGAGGGGATAACCTCCACTCAGGCCAAGCAGCTTGGTTTGGGAGGGCTGGTCATGGGATATAACGCTTCTGGTGTTATTGGGGCTGTGGGCGAAATAGACACCGGTCACCGCCTTAATGCATCAGGAGCAAGTACTCCCGGAAGCGAAACTTCTGTGGAGAGTTTTGTGAACGGACAGAAACCCGCCGGAGAATGGCATGCTGTGGCGAAGGACAGCTGGACAGGGGCTGGTCCAGTGAATGTAGGGCTGGTTAATAACGCCATCAAAAGTGTCCGGATTATCAAAAAAGGCTATGTGACTGGTGTTCTCTTGCCTGAAGAGGTGATGAATAAAGCAGAGTACAAAGCAATGCGGCAGGCTTTCGATTCCCTTCCACTGGCAAAAC |
| NE3 | U | LEE0134 | TACTCTGCTTTATTCATCACCTCTTCAGGCAAGAGAACACCAGTCACATAGCCTTTTTTGATAATCCGGACACTTTTGATGGCGTTATTAACCAGCCCTACATTCACTGGACCAGCCCCTGTCCAGCTGTCCTTCGCCACAGCATGCCATTCTCCGGCGGGTTTCTGTCCGTTCACAAAACTCTCCACAGAAGTTTCGCTTCCGGGAGTACTTGCTCCTGATGCATTAAGGCGGTGACCGGTGTCTATTTCGCCCACAGCCCCAATAACACCAGAAGCGTTATATCCCATGACCAGCCCTCCCAAACCAAGCTGCTTGGCCTGAGTGGAGGTTATCCCCTCAATGGTGACCTGCATAACACCAGAAGGAGCAATACTTCCCAGAATCATTTTTGCTTCAGGATTCATTTT |
