# Supplementary material for: Acacia Fiber Protects the Gut from Extended-Spectrum Beta-Lactamase (ESBL)-Producing Escherichia coli Colonization Enabled by Antibiotics
Source: mSphere. 2022 May 18;7(3):e00071-22. doi: 10.1128/msphere.00071-22 (PMC9241499; doi:10.1128/msphere.00071-22)
Supplement: TABLE S2 [file msphere.00071-22-s0002.docx]

**Table S2: Significant (adjusted *p* < 0.05) OTU abundance contrasts between time points per bacterial family**

**References**

1. [McMurdie PJ, Holmes S. 2013. phyloseq: an R package for reproducible interactive analysis and graphics of microbiome census data. PLoS One 8:e61217.](http://paperpile.com/b/ItHLdd/zcbUT)
2. [Love MI, Huber W, Anders S. 2014. Moderated estimation of fold change and dispersion for RNA-seq data with DESeq2. Genome Biol 15:550.](http://paperpile.com/b/Kz5gN1/lqUlZ)
