# Supplementary material for: Acacia Fiber Protects the Gut from Extended-Spectrum Beta-Lactamase (ESBL)-Producing Escherichia coli Colonization Enabled by Antibiotics
Source: mSphere. 2022 May 18;7(3):e00071-22. doi: 10.1128/msphere.00071-22 (PMC9241499; doi:10.1128/msphere.00071-22)
Supplement: TABLE S3 [file msphere.00071-22-s0003.docx]

| **Table S3: Summary of Primers** | | | | | |
| --- | --- | --- | --- | --- | --- |
| **Primer Name** | **Target** | **Primer Direction** | **5’-3’ Sequence** | **Product Size (bp)** | **Source** |
| MM0421 | *bla*_CTX-M-15_ ESBL gene | Forward | TTGTTAGGAAGTGTGCCGCT | 302 | This study |
| MM0422 | *bla*_CTX-M-15_ ESBL gene | Reverse | ATCGTCCCATTGACGTGCTT | 302 | This study |
| MM0429 | JJ1886 | Forward | ACATTTCGCCTTACCCGTCTT | 177 | This study |
| MM0430 | JJ1886 | Reverse | TCCACCCCGGGATTTAGGTTT | 177 | This study |
| MM0463 | Universal 16S rRNA | Forward | AAACTCAAAKGAATTGACGG | 136 | REF(1) |
| MM0464 | Universal 16S rRNA | Reverse | CTCACRRCACGAGCTGAC | 136 | REF(1) |
| LEE0035 | ZEO resistance cassette | Forward | CAAGTTGACCAGTGCCGTTC | 250 | REF(2) |
| LEE0036 | ZEO resistance cassette | Reverse | GTTCGTGGACACGACCTCC | 250 | REF(2) |
| LEE0127 | Colicin M activity gene | Forward | GCTTACCACTTCGCAAAACC | 429 | REF(3) |
| LEE0128 | Colicin M activity gene | Reverse | GAGCGACTCTCCGATAATGC | 429 | REF(3) |
| LEE0097 | Colicin B activity gene | Forward | AAGAAAATGACGAGAAGACG | 493 | REF(4) |
| LEE0098 | Colicin B activity gene | Reverse | GAAAGACCAAAGGCTATAAGG | 493 | REF(4) |
| LEE0133 | Colicin U activity gene | Forward | TGATTGCTGCGAGAAAAATG | 485 | REF(3) |
| LEE0134 | Colicin U activity gene | Reverse | TCTGACAGCCTCTCCCTGTT | 485 | REF(3) |
| LEE0135 | Colicin Y activity gene | Forward | GCAGGCAGAAAAGAACAAGG | 477 | REF(4) |
| LEE0136 | Colicin Y activity gene | Reverse | CGGACGTTATTTGCCTTCAT | 477 | REF(4) |

**References**

1. Maeusli M, Lee B, Miller S, Reyna Z, Lu P, Yan J, Ulhaq A, Skandalis N, Spellberg B, Luna B. 2020. Horizontal Gene Transfer of Antibiotic Resistance from *Acinetobacter baylyi* to *Escherichia coli* on Lettuce and Subsequent Antibiotic Resistance Transmission to the Gut Microbiome. mSphere 5.
2. Yang Y-W, Chen M-K, Yang B-Y, Huang X-J, Zhang X-R, He L-Q, Zhang J, Hua Z-C. 2015. Use of 16S rRNA Gene-Targeted Group-Specific Primers for Real-Time PCR Analysis of Predominant Bacteria in Mouse Feces. Appl Environ Microbiol 81:6749–6756.
3. Smajs D, Micenková L, Smarda J, Vrba M, Sevčíková A, Vališová Z, Woznicová V. 2010. Bacteriocin synthesis in uropathogenic and commensal *Escherichia coli*: colicin E1 is a potential virulence factor. BMC Microbiol 10:288.
4. Gordon DM, O’Brien CL. 2006. Bacteriocin diversity and the frequency of multiple bacteriocin production in *Escherichia coli*. Microbiology 152:3239–3244.
