# Supplementary material for: Acacia Fiber Protects the Gut from Extended-Spectrum Beta-Lactamase (ESBL)-Producing Escherichia coli Colonization Enabled by Antibiotics
Source: mSphere. 2022 May 18;7(3):e00071-22. doi: 10.1128/msphere.00071-22 (PMC9241499; doi:10.1128/msphere.00071-22)
Supplement: TABLE S4 [file msphere.00071-22-s0004.docx]

| **Table S4: Summary of bacterial strains and plasmids used in this study** | | |
| --- | --- | --- |
| **Strains** | **Description** | **Reference** |
| *E. coli* JJ1886 | Blood and urine clinical isolate. ESBL. ST131, CTX-M-15-producing *H*30Rx sublineage. | REF(1). |
| *E. coli* NE1 | Murine gut *E. coli* isolated from feces. | This study. |
| *E. coli* NE3 | Murine gut *E. coli* isolated from feces. | This study. |
| *E. coli* NEC | Combined *E. coli* NE1 and NE3. | This study. |
| *E. coli* NE1 ΔB | *E. coli* NE1 colicin B activity (*cba*) gene knock out with zeoR insertion. | This study. |
| *E. coli* NE1 Δ(YBM) | *E. coli* NE1 colicin Y, B, M activity (*cya*, *cba*, *cma*) gene knock out with zeoR insertion. | This study. |
| *E. coli* NE1 ΔM | *E. coli* NE1 colicin M activity (*cma*) gene knock out with zeoR insertion. | This study. |
| *E. coli* NE1 ΔM comp. | *E. coli* NE1 ΔM transformed with pTO4. | This study. |
| *S. aureus* LAC | USA300 methicillin-resistant *S. aureus* clinical isolate | REF(2). |
| **Plasmids** | **Description** | **Reference** |
| pKM200 | Backbone plasmid for lambda-red mediated homologous recombination. CAM resistance. | REF(3). |
| pTO4 | Encodes colicin M activity (*cma*) and immunity (*cmi*) genes. | Gift from D. Mengin-Lecreulx. Original source REF(4). |

**References**

1. Owens RC Jr, Johnson JR, Stogsdill P, Yarmus L, Lolans K, Quinn J. 2011. Community transmission in the United States of a CTX-M-15-producing sequence type ST131 *Escherichia coli* strain resulting in death. J Clin Microbiol 49:3406–3408.
2. [Lin L, Ibrahim AS, Xu X, Farber JM, Avanesian V, Baquir B, Fu Y, French SW, Edwards JE Jr, Spellberg B. 2009. Th1-Th17 cells mediate protective adaptive immunity against *Staphylococcus aureus* and *Candida albicans* infection in mice. PLoS Pathog 5:e1000703.](http://paperpile.com/b/9mrvLq/VkMx)
3. [Murphy KC, Campellone KG. 2003. Lambda Red-mediated recombinogenic engineering of enterohemorrhagic and enteropathogenic *E. coli*. BMC Mol Biol 4:11.](http://paperpile.com/b/9mrvLq/wP0Q)
4. [Ölschläger T, Schramm E, Braun V. 1984. Cloning and expression of the activity and immunity genes of colicins B and M on ColBM plasmids. Mol Gen Genet 196:482–487.](http://paperpile.com/b/9mrvLq/jdT7)
