# Supplementary material for: Acacia Fiber Protects the Gut from Extended-Spectrum Beta-Lactamase (ESBL)-Producing Escherichia coli Colonization Enabled by Antibiotics
Source: mSphere. 2022 May 18;7(3):e00071-22. doi: 10.1128/msphere.00071-22 (PMC9241499; doi:10.1128/msphere.00071-22)
Supplement: TABLE S5 [file msphere.00071-22-s0005.docx]

| **Table S5: Colicin Knockout *E. coli* Mutant DNA Sequences with Zeocin Insertion** | | | |
| --- | --- | --- | --- |
| **Zeocin Insertion Construct** | | **Length (bp)** | **DNA Sequence^a^** |
|  |  | 776 | **taacgcttacaatttcctgatgcggtattttctccttacgcatctgtgcggtatttcacaccgcatacaggtggcacttttcggggaaatgtgcgcggaacccctatttgtttatttttctaaatacattcaaatatgtatccgctcatgagacaataaccctgataaatgcttcaataatagcacgtgaggagggccaccATGGCCAAGTTGACCAGTGCCGTTCCGGTGCTCACCGCGCGCGACGTCGCCGGAGCGGTCGAGTTCTGGACCGACCGGCTCGGGTTCTCCCGGGACTTCGTGGAGGACGACTTCGCCGGTGTGGTCCGGGACGACGTGACCCTGTTCATCAGCGCGGTCCAGGACCAGGTGGTGCCGGACAACACCCTGGCCTGGGTGTGGGTGCGCGGCCTGGACGAGCTGTACGCCGAGTGGTCGGAGGTCGTGTCCACGAACTTCCGGGACGCCTCCGGGCCGGCCATGACCGAGATCGGCGAGCAGCCGTGGGGGCGGGAGTTCGCCCTGCGCGACCCGGCCGGCAACTGCGTGCACTTCGTGGCCGAGGAGCAGGACTGAcacgtgctaaaacttcatttttaatttaaaaggatctaggtgaagatcctttttgataatctcatgaccaaaatcccttaacgtgagttttcgttccactgagcgtcagaccccgtagaaaagatcaaaggatcttcttgagatcctttttttctgcgcgtaatctgctgcttgcaaacaaaaaaaccaccgctaccagc** |
| ***E. coli* Isolate** | **Target Colicin** | **Length (bp)** | **DNA Sequence^a^** |
| NE1 | B | 1215 | ATCATTAGTGTCGGTGACAAAGTCGGGGAATATCTTGGAGATAAATACAAGGCGCTTTCCCGTGAAATTGCAGAGAATATAAATAATTTTCAGGGAAAAACGATTCGTAGTTATGATGATGCAATGTCTTCCATTAATAAGTTAATGGCTAACCCCAGCCTTAAAATAAATGCAACGGACAAAGAAGCCATTGTGAATGCGTGGAAAGCATTTAATGC**taacgcttacaatttcctgatgcggtattttctccttacgcatctgtgcggtatttcacaccgcatacaggtggcacttttcggggaaatgtgcgcggaacccctatttgtttatttttctaaatacattcaaatatgtatccgctcatgagacaataaccctgataaatgcttcaataatagcacgtgaggagggccaccATGGCCAAGTTGACCAGTGCCGTTCCGGTGCTCACCGCGCGCGACGTCGCCGGAGCGGTCGAGTTCTGGACCGACCGGCTCGGGTTCTCCCGGGACTTCGTGGAGGACGACTTCGCCGGTGTGGTCCGGGACGACGTGACCCTGTTCATCAGCGCGGTCCAGGACCAGGTGGTGCCGGACAACACCCTGGCCTGGGTGTGGGTGCGCGGCCTGGACGAGCTGTACGCCGAGTGGTCGGAGGTCGTGTCCACGAACTTCCGGGACGCCTCCGGGCCGGCCATGACCGAGATCGGCGAGCAGCCGTGGGGGCGGGAGTTCGCCCTGCGCGACCCGGCCGGCAACTGCGTGCACTTCGTGGCCGAGGAGCAGGACTGAcacgtgctaaaacttcatttttaatttaaaaggatctaggtgaagatcctttttgataatctcatgaccaaaatcccttaacgtgagttttcgttccactgagcgtcagaccccgtagaaaagatcaaaggatcttcttgagatcctttttttctgcgcgtaatctgctgcttgcaaacaaaaaaaccaccgctaccagc**TGAGGATATGGGGAATAAATTTGCTGCGTTGGGTAAAACGTTCAAAGCAGCAGATTATGCAATAAAGGCAAACAACATCAGGGAGAAGAGTATTGAGGGTTACCAGACTGGTAACTGGGGGCCATTAATGCTGGAAGTCGAGTCCTGGGTTATCAGTGGGATGGCATCTGCTGTAGCTCTTAGTTTGTTTTCTTTGACATTAGGCTCGGCCCTTATAGCCT |
| NE1 | M | 1143 | CATGAATATGAAACAAATGAGCGGTAATGTCACTACACCAATTGTGGCGCTTGCTCACTATTTATGGGGTAATGGCGCTGAAAGGAGCGTTAATATCGCCAACATTGGTCTTAAAATTTCCCCTATGAAAATTAATCAGATAAAAGACATTATAAAATCTGGTGTAGTAGGTACATTCC**taacgcttacaatttcctgatgcggtattttctccttacgcatctgtgcggtatttcacaccgcatacaggtggcacttttcggggaaatgtgcgcggaacccctatttgtttatttttctaaatacattcaaatatgtatccgctcatgagacaataaccctgataaatgcttcaataatagcacgtgaggagggccaccATGGCCAAGTTGACCAGTGCCGTTCCGGTGCTCACCGCGCGCGACGTCGCCGGAGCGGTCGAGTTCTGGACCGACCGGCTCGGGTTCTCCCGGGACTTCGTGGAGGACGACTTCGCCGGTGTGGTCCGGGACGACGTGACCCTGTTCATCAGCGCGGTCCAGGACCAGGTGGTGCCGGACAACACCCTGGCCTGGGTGTGGGTGCGCGGCCTGGACGAGCTGTACGCCGAGTGGTCGGAGGTCGTGTCCACGAACTTCCGGGACGCCTCCGGGCCGGCCATGACCGAGATCGGCGAGCAGCCGTGGGGGCGGGAGTTCGCCCTGCGCGACCCGGCCGGCAACTGCGTGCACTTCGTGGCCGAGGAGCAGGACTGAcacgtgctaaaacttcatttttaatttaaaaggatctaggtgaagatcctttttgataatctcatgaccaaaatcccttaacgtgagttttcgttccactgagcgtcagaccccgtagaaaagatcaaaggatcttcttgagatcctttttttctgcgcgtaatctgctgcttgcaaacaaaaaaaccaccgctaccagc**CTGTTTCTACAAAGTTCACACATGCCACTGGTGATTATAATGTTATTACCGGTGCATATCTTGGTAATATCACACTGAAAACAGAAGGTACTTTAACTATCTCTGCCAATGGCTCCTGGACTTACAATGGCGTTGTTCGTTCATATGATGATAAATACGATTTTAACGCCAGCACTCACCGTGGCATT |
| NE1 | Y | 1186 | AGCTGAAGCTGAAAAAGCGGCTGCTGAAGCAAAAGCAAAAGCTGAAGCTGAAAAAGCCAGAAAGGAAGCTGAAGCAAAAGCAAATAACGAGAAAGCTGTTCTGACAAAAGCCAGTGAAATTATCATTAGTGTGGGTGATAAGGTCGGAGAATATCTTGGCGATAAATATAAAGCTCTTTCTCGTGAGATAGCAGGTAATATCAA**taacgcttacaatttcctgatgcggtattttctccttacgcatctgtgcggtatttcacaccgcatacaggtggcacttttcggggaaatgtgcgcggaacccctatttgtttatttttctaaatacattcaaatatgtatccgctcatgagacaataaccctgataaatgcttcaataatagcacgtgaggagggccaccATGGCCAAGTTGACCAGTGCCGTTCCGGTGCTCACCGCGCGCGACGTCGCCGGAGCGGTCGAGTTCTGGACCGACCGGCTCGGGTTCTCCCGGGACTTCGTGGAGGACGACTTCGCCGGTGTGGTCCGGGACGACGTGACCCTGTTCATCAGCGCGGTCCAGGACCAGGTGGTGCCGGACAACACCCTGGCCTGGGTGTGGGTGCGCGGCCTGGACGAGCTGTACGCCGAGTGGTCGGAGGTCGTGTCCACGAACTTCCGGGACGCCTCCGGGCCGGCCATGACCGAGATCGGCGAGCAGCCGTGGGGGCGGGAGTTCGCCCTGCGCGACCCGGCCGGCAACTGCGTGCACTTCGTGGCCGAGGAGCAGGACTGAcacgtgctaaaacttcatttttaatttaaaaggatctaggtgaagatcctttttgataatctcatgaccaaaatcccttaacgtgagttttcgttccactgagcgtcagaccccgtagaaaagatcaaaggatcttcttgagatcctttttttctgcgcgtaatctgctgcttgcaaacaaaaaaaccaccgctaccagc**AAACTTTCAAGGTAAGACGATCCGTAGTTATGATGAGGCGATGGCTTCTGTCAATAAACTGATGGCTAATCCTGATCTTAAAATTAATGCTGCAGACAGGGATGTCATTGTGAATGCCTGGAAAGCATTTGATGCAGAGGATATGGGGAATAAGTTTGCCGCGCTGGGTAAGACATTTAAAGCCGCAGATTATGTGATGAAGGCAA |
| ^a^Nucleotides in bold represent the zeocin insertion construct. Nucleotides that are not in bold represent the respective colicin activity gene sequence. | | | |
